# PARP Inhibitors in Ovarian Cancer: Resistance Mechanisms, Clinical Evidence, and Evolving Strategies

**DOI:** 10.3390/biomedicines13051126

**Published:** 2025-05-06

**Authors:** Shant Apelian, Antons Martincuks, Michelle Whittum, Maya Yasukawa, Lindsey Nguy, Begum Mathyk, Vaagn Andikyan, Matthew L. Anderson, Thomas Rutherford, Mihaela Cristea, Daphne Stewart, Adrian Kohut

**Affiliations:** 1 Department of Obstetrics and Gynecology, Division of Gynecologic Oncology, University of South Florida, Tampa, FL 33620, USA; michellwhittum@usf.edu (M.W.); myasukawa@usf.edu (M.Y.); lindseynguy@usf.edu (L.N.); abegum@usf.edu (B.M.); andikyan@usf.edu (V.A.); mlander5@usf.edu (M.L.A.); rutherftom@gmail.com (T.R.); akohut@usf.edu (A.K.); 2 Division of Gynecologic Oncology, Tampa General Hospital Cancer Institute, Tampa, FL 33620, USA; 3 Department of Obstetrics, Gynecology & Reproductive Sciences, Yale School of Medicine, New Haven, CT 06510, USA; 4 Department of Immuno-Oncology, City of Hope National Medicinal Center, Duarte, CA 91010, USA; amartincuks@coh.org; 5 Regeneron Pharmaceuticals, Tarrytown, NY 10591, USA; mihaela.cristea@regeneron.com; 6 Department of Medicine, Division of Medical Oncology, USC Norris Comprehensive Cancer Center and Keck School of Medicine, Los Angeles, CA 90089, USA; daphnest@usc.edu

**Keywords:** PARP inhibitors, platinum-based chemotherapy, drug resistance, ovarian neoplasms

## Abstract

The introduction of poly (ADP-ribose) polymerase inhibitors (PARPi) into the management of ovarian cancer has transformed the treatment landscape for patients affected by this malignancy. However, as the use of PARPi expands into both frontline maintenance and recurrence settings, the emergence of drug resistance has become a significant clinical challenge in the treatment of these patients. Although platinum-based chemotherapy (PBC) and PARPi act through different mechanisms—PBC causes DNA damage while PARPi blocks its repair—both depend on the integrity of DNA damage repair (DDR) pathways, leading to overlapping mechanisms of resistance. Here, we review the key resistance mechanisms shared by PARPi and PBC, and then we discuss their clinical implications in the management of patients with ovarian cancer. We also examine clinical rationale supporting the hypothesis that prior PARPi exposure may reduce the efficacy of subsequent PBC in patients experiencing a disease recurrence. Furthermore, we review preliminary clinical data assessing the potential role of PARPi retreatment in patients who have previously progressed on PARPis.

## 1. Introduction

The management of ovarian cancer has undergone considerable innovation over recent years due to the successful integration of poly (ADP- ribose) polymerase (PARP) inhibitors (PARPis) into contemporary therapeutic strategies. Prior to this, the pharmacological standard of care for ovarian cancer primarily involved platinum-based chemotherapy (PBC). PARPis are targeted agents that exploit the dependence of rapidly dividing cells on proficient deoxyribonucleic acid (DNA) repair mechanisms for survival and proliferation. Given the exponential growth of these cells, they rely on an array of DNA repair mechanisms that enable them to maintain a continuous state of cellular division [1]. Critical DNA repair mechanisms include single-strand and double-strand break (SSB and DSB) DNA recognition and repair, nonhomologous end joining (NHEJ), homologous recombination (HR), base excision repair (BER), and nucleotide excision repair (NER) [1,2,3,4,5]. The PARP enzyme family plays a vital role in these DNA repair mechanisms, making them attractive targets in developing cancer therapeutics [5,6,7,8,9,10,11,12,13,14,15].

Early studies on PARPis highlighted the therapeutic potential of these agents in ovarian cancer, particularly due to the high prevalence of germline mutations in critical DNA repair genes such as *BRCA1* and *BRCA2* [16]. Subsequent studies showed similar benefits in patients with somatic *BRCA* mutations or *BRCA* wild-type tumors exhibiting homologous recombination deficiency (HRD), thereby expanding the population of patients with ovarian cancer likely to respond to PARPi [17,18,19,20,21]. These findings support the use of *BRCA* and HR status as biomarkers predictive of response to PARPi [18].

The initial FDA approvals of PARPis, including olaparib, niraparib, and rucaparib, for ovarian cancer were granted based on phase III clinical trials demonstrating significant efficacy in maintaining remission in recurrent platinum-sensitive ovarian cancer [22,23,24]. The most significant benefit was observed in patients with germline *BRCA* mutations [22,23,24]. Given the success of these agents in the recurrent setting, PARPis were subsequently evaluated in earlier-stage disease through additional phase III trials, which showed improved survival outcomes. These findings led to FDA approvals for the use of PARPi in the frontline maintenance setting, including olaparib for patients with *BRCA*-mutated platinum-responsive disease, olaparib plus bevacizumab for patients with HRD-positive, platinum-responsive disease, and niraparib for all patients with platinum-responsive disease, regardless of biomarker status [25,26,27,28].

The successful integration of PARPis in the management of high-grade serous ovarian cancer has, therefore, provided significant clinical benefits and reshaped the therapeutic landscape for these patients. However, it has also introduced new challenges in managing patients with disease recurrence after the use of PARPis. In this review, we explored the complexities of systemic therapy following relapse and provided an overview of the ongoing efforts to address these emerging challenges.

## 2. Shared Mechanisms of Resistance to PARP Inhibitors and Platinum-Based Chemotherapy

**Figure 1:**
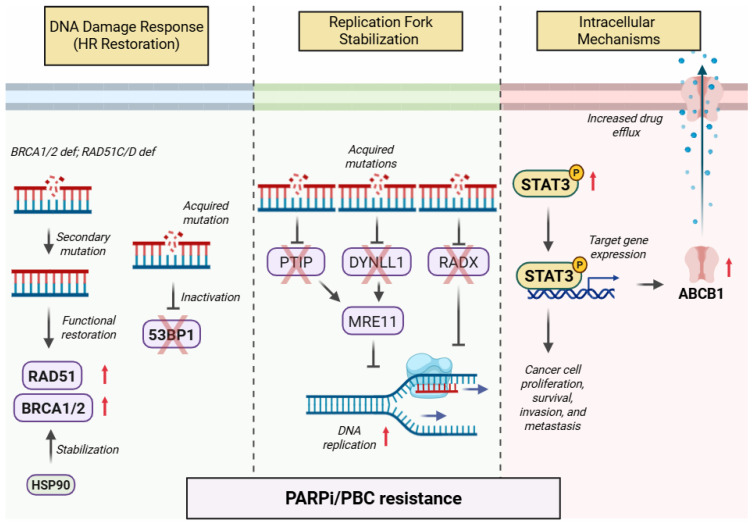
Mechanisms of resistance to PARP inhibitors and platinum-based therapies in cancer cells. This figure outlines the cellular mechanisms contributing to resistance against PARP inhibitors and platinum-based chemotherapy. It is divided into three key categories: DNA damage response (illustrated here by homologous recombination restoration), replication fork stabilization, and intracellular mechanisms such as an enhanced expression of drug efflux transporters and increased activation of oncogenic signaling pathways. ABCB1, ATP-binding cassette subfamily B member 1; BRCA1/2, breast cancer gene 1/2; DNA, deoxyribonucleic acid; DYNLL1, dynein light chain LC8-Type 1; HR, homologous recombination; HSP90, heat shock protein 90; MRE11, MRE11 homolog; PARPi, PARP inhibitor; PTIP, PAX interacting protein 1; RAD51, RAD51 recombinase; RAD51C/D, RAD51 homolog C/D; RADX, regulator of DNA replication fork stability; STAT3, signal transducer and activator of transcription 3; and 53BP1, tumor suppressor p53 binding protein 1.

With the expanding use of PARPis and PBC in ovarian cancer, resistance has emerged as a major barrier to sustained efficacy. Rather than isolated mechanisms, these resistance mechanisms reflect a spectrum of adaptive processes that allow tumor cells to evade DNA-damaging agents [29,30]. They are broadly classified into three biological domains: DNA damage repair response, replication fork stabilization, and intracellular adaptations such as increased drug efflux (Figure 1).

### 2.1. DNA Damage Repair Response

**Figure 2:**
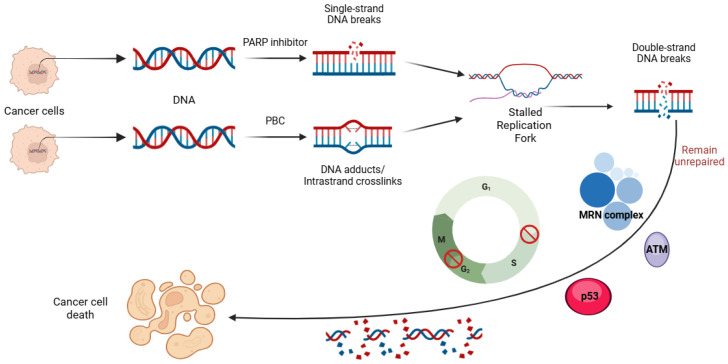
Mechanism of action of PARP inhibitors and platinum-based chemotherapy. PARP inhibition blocks the repair of single-strand DNA breaks (SSBs), leading to replication fork stalling and collapse during the S-phase, which converts SSBs into double-strand breaks (DSBs). PBCs, on the other hand, introduce DNA adducts and intrastrand crosslinks, similarly stalling replication forks and producing DSBs. The resulting DSBs activate the MRN complex and ATM kinase, initiating the DNA damage response and triggering p53-mediated cell cycle arrest at both the G1/S and G2/M checkpoints if they remain unrepaired. These events eventually lead to genomic instability and cancer cell death. PARP, poly (ADP-ribose) polymerase; PBC, platinum-based chemotherapy; MRN, MRE11-RAD50-NBS1 complex; and ATM, ataxia telangiectasia mutated kinase.

**Figure 3:**
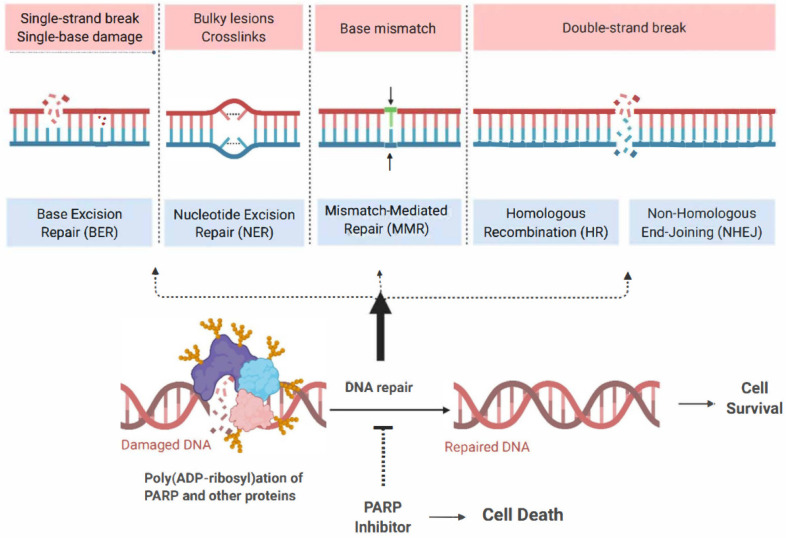
Pathways of DNA damage response and the impact of PARP inhibition on cellular outcome. This figure illustrates various DNA damage types and their corresponding damage response pathways, highlighting the role of PARP in maintaining genomic stability and cell survival. Inhibition of PARP prevents these pathways, leading to the accumulation of damaged DNA and subsequent cell death. DNA, deoxyribonucleic acid; PARP, poly (ADP-Ribose) polymerase.

PBCs exert their cytotoxic effects by covalently binding to DNA, forming a series of mono-adducts that eventually lead to interstrand DNA crosslinks [31,32]. If these interlinks are left unrepaired, they can disrupt DNA synthesis by delaying the replication fork and triggering apoptosis (Figure 2). Tumors with HRD are particularly susceptible, as they lack proficient DNA repair mechanisms to resolve these lesions [31,32]. The critical DNA damage response (DDR) pathways involved in repairing platinum-induced DNA crosslinks, including SSB, DSB, NHEJ, and HR [1,2,3,4,5], are also key targets of PARPis (Figure 3) [32,33]. By blocking DDR pathways from repairing damaged DNA, these agents promote the conversion of SSBs to lethal DSBs, ultimately leading to cancer cell death [34,35]. However, restoring DDR function in cancer cells could drive resistance to both PBC and PARPi.

Since these agents rely on the intracellular DDR pathways, several overlapping molecular mechanisms contributing to primary and acquired resistance to both platinum-based therapies and PARPi have been identified.

One of the most well-characterized mechanisms by which cancer cells can restore HR proficiency and evade DNA damage induced by PBC and PARPi is the acquisition of reversion mutations in key genes involved in the HR repair pathway. Reversion of inhibitory *BRCA1/2* mutations as a mechanism of acquired resistance to either PARPi or cisplatin was initially shown using *BRCA2*-deficient ovarian and pancreatic cancer cells [36,37]. These reversion mutations are secondary in the pathogenic mutations in key HR repair genes, such as pathogenic *BRCA1/2* mutations, which modify the original *BRCA1/2* alterations, restoring in-frame transcription and producing partially functional protein products [38]. Reversion mutations can arise in both germline and somatic *BRCA1/2* mutations, ultimately leading to acquired resistance against DNA-damaging agents such as PARPi and PBC [38,39,40,41].

Studies have also identified *BRCA*-independent mechanisms of HR restoration, further supporting the role of secondary reversion mutations in promoting resistance to both PARPi and PBC. Secondary mutations in *RAD51C* and *RAD51D*, as well as inactivation of *53BP1*, have been shown to rescue HR function [42,43]. Johnson et al. described two such mechanisms using a *BRCA1* BRCA C-terminal domain-mutated breast cancer cell line: heat shock protein 90 (HSP90)-mediated stabilization of a mutant BRCA1 protein and mutations in the tumor protein p53 binding protein 1 (TP53BP1) that restore DNA end resection and facilitate RAD51 filament formation, key components of the HR repair pathway [44].

### 2.2. Replication Fork Stabilization

Restoration of DNA replication fork stability is another key mechanism driving both PARPi and PBC resistance. For example, the loss of MRE11 nuclease recruitment to stalled replication forks has been shown to confer PARPi resistance in *BRC1/2*-deficient cells by stabilizing replication forks [45]. Similarly, depletion of DYNLL1 or RADX, essential factors that prevent aberrant fork protection, reduces the cytotoxic effects of both PARPi and PBC in *BRCA*-deficient cancer cells, showcasing the role of replication fork stability in therapy resistance [46,47].

### 2.3. Intracellular Mechanisms

Additional intracellular mechanisms contributing to resistance against both PARPi and PBC include upregulated expression of drug efflux transporters and increased activation of oncogenic signaling pathways. One of the earliest identified resistance mechanisms was the upregulation of the ABCB1 (MDR1, P-glycoprotein) drug efflux transporter, which reduces intracellular drug accumulation [48] and has been observed in both PARPi- and platinum-resistant ovarian cancer cells [49]. Additionally, constitutive activation of the oncogenic STAT3 signaling pathway has been demonstrated across several studies to promote platinum resistance by enhancing cancer cell survival and proliferation [50,51,52]. We have recently observed increased STAT3 activity in PARPi-resistant ovarian cancer cells [53].

Beyond these mechanisms, epigenetic dysregulation has emerged as a multifaceted contributor to therapeutic resistance. Epigenetic changes—including DNA methylation, histone modifications, and chromatin remodeling—can drive resistance by influencing gene expression across all three major domains: DDR, replication fork stability, and intracellular signaling. For instance, promoter demethylation or histone acetylation can restore homologous recombination proficiency via re-expression of silenced HR genes such as BRCA1, directly counteracting the effects of PARP inhibition and platinum-induced DNA damage [54,55]. In the replication context, chromatin compaction or nucleosome repositioning may enhance fork protection and limit access to endonucleases, reducing replication-associated cytotoxicity [56]. Furthermore, there are epigenetic alterations that can exacerbate drug efflux and resistance to apoptosis [57]. These epigenetic alterations are dynamic and reversible, offering potential therapeutic avenues to resensitize tumors to both PBC and PARPi [57,58].

These findings underscore the interplay between PARPi and PBC resistance mechanisms and highlight the potential for cross-resistance between these two therapeutic classes. A deeper understanding of these biological processes is essential for developing novel, more effective treatment strategies to overcome resistance and improve patient outcomes.

## 3. Placebo-Controlled Trials Evaluating the Role of PARP Inhibitors in Ovarian Cancer

Table 1 summarizes clinical trials evaluating the efficacy of PARPis (olaparib, niraparib, and rucaparib) in the maintenance and treatment of recurrent ovarian cancers. It details study settings, inclusion criteria, *BRCA* and HR statuses, and their endpoints, including the primary and secondary ones.

### 3.1. Frontline Treatment

PARPis have not yet been broadly adopted as concurrent agents with frontline PBC, primarily due to overlapping toxicities [59]. The most notable study in this setting is the VELIA trial, which investigated veliparib in combination with chemotherapy and as maintenance therapy [60]. A statistically significant improvement in median progression-free survival (PFS) was observed across all biomarker-defined subgroups, including patients with *BRCA* mutations (34.7 vs. 22.0 months; HR 0.44, 95% CI of 0.28 to 0.68), HRD-positive tumors (31.9 vs. 20.5 months; HR 0.57, 95% CI of 0.43 to 0.76), and the intention-to-treat population (23.5 vs. 17.3 months; HR 0.68, 95% CI of 0.56 to 0.83). Importantly, the benefit was observed only when veliparib was continued into maintenance; no survival advantage was seen with veliparib use during the induction phase alone. Therefore, the use of PARPis with frontline chemotherapy is limited by toxicity and minimal added benefit, with their primary value lying in their continuation as maintenance therapy rather than as a standalone frontline treatment.

### 3.2. Frontline Maintenance

The most consistent and significant clinical impact of PARPis has been observed in the frontline maintenance setting, particularly in those with germline *BRCA1/2*, somatic *BRCA1/2*, or germline *BRCA1/2* wild-type HR-deficient biomarker statuses. Several phase III trials have shown clinical benefits with niraparib (PRIMA/ENGOT-OV26/GOG-3012), olaparib (SOLO1/GOG3004 and PAOLA-1/ENGOT-ov25), and rucaparib (ATHENA-MONO/GOG-3020/ENGOT-ov45) following initial response to PBC [26,27,28,61,62,63,64]. These findings were further confirmed by mature OS results from the PRIMA and PAOLA-1 trials, which indicated significantly longer median OS among patients with homologous recombination-deficient (HRD) ovarian cancer who received PARPi [65,66]. However, the 7-year follow-up data from the SOLO1/GOG-3004 trial showed no significant difference in OS between patients with *BRCA*-mutated ovarian cancer who received frontline maintenance olaparib and those who received a placebo [63]. Additionally, final safety results from the ATHENA-MONO trial confirmed a manageable safety profile for rucaparib, though final efficacy results are still pending [67]. Patients with *BRCA1/2* wild-type, HR-proficient biomarker statuses experienced less benefit from PARPis, with only niraparib and rucaparib demonstrating statistically significant survival advantages [27,61].

### 3.3. Recurrent Treatment

PARPis have also been tested as active treatment agents in the setting of platinum-sensitive recurrent ovarian cancer. Key trials include SOLO-3 and ARIEL-4, both limited to *BRCA*-mutated populations [68,69,70,71,72]. SOLO-3 demonstrated a median PFS of 13.2 months with olaparib vs. 8.5 months with chemotherapy (HR 0.62, 95% CI of 0.43 to 0.91), though the OS difference (34.9 vs. 32.9 months) was not statistically significant. ARIEL-4, which compared rucaparib with chemotherapy in patients with *BRCA*-mutated recurrent disease, showed a modest PFS benefit (7.4 vs. 5.7 months; HR 0.67, 95% CI of 0.52 to 0.86) [68,69]. However, it raised concern due to a non-statistically significant trend toward worse OS in the rucaparib group (19.4 vs. 25.4 months; HR 1.31, 95% CI of 1.00 to 1.73) [68,69].

Therefore, although PARPis offer an alternative to chemotherapy in recurrent *BRCA*-mutated disease by improving PFS, inconsistent OS results suggest a need for careful patient selection and further study.

### 3.4. Recurrent Maintenance

PARPis have shown consistent PFS benefit in the maintenance setting following a response to platinum-based therapy for recurrent disease, particularly in patients with positive biomarker statuses. Trials include NOVA (niraparib), NORA (niraparib, Asian cohort), ARIEL-3 (rucaparib), and SOLO2 (olaparib) [23,73,74,75,76,77,78,79]. However, in this setting, no PARPi has yet demonstrated a significant OS benefit in the _g_BRCA_wt/_HRp population [23,73,74,76,77]. The only PARPi to show an OS benefit in the recurrent maintenance setting within a phase III trial was olaparib in the SOLO2 trial [78,79], which included only patients with germline or somatic *BRCA* or *BRCA* mutation biomarker statuses. In SOLO2, olaparib significantly improved both PFS (19.1 vs. 5.5 months; HR 0.30, 95% CI of 0.22 to 0.41) and OS (51.7 vs. 38.8 months; HR 0.30, 95% CI of 0.22 to 0.41) in *BRCA*-mutated patients. OS results in HR-proficient populations were less favorable, with some trials reporting non-significant or negative trends (e.g., ARIEL-3: OS HR for HRp population = 1.15, 95% CI of 0.78 to 1.70) [23,73,74,76,77].

**Table 1:** Summary of clinical trials evaluating PARP inhibitors in patients with ovarian cancer.

Setting	Drug	Study	Inclusion Criteria	*BRCA*/HR Status	Methods	Primary Endpoint	Pertinent Secondary Endpoints
***Frontline Maintenance***	Niraparib	PRIMA [27,61]	High-grade epithelial histology Stage III or IV Primary CRS or Interval CRS Complete or partial response after PBC (bevacizumab not permitted)	_g_*BRCA*_m_ _g_*BRCA*_wt_/HRd _g_*BRCA*_wt_/HRp	Phase 3 2:1 ratio to receive niraparib 300 mg or placebo once daily for up to 36 months.	*Median PFS:* *ITT Population:* niraparib: 13.8 months placebo: 8.2 months HR, 0.62; 95% CI, 0.50 to 0.76 *HRd Population:* niraparib: 21.9 months placebo 10.4 months HR, 0.43; 95% CI, 0.31 to 0.59 *HRp Population:* niraparib: 8.1 months placebo: 5.4 months HR, 0.68; 95% CI, 0.49 to 0.94	*24-Month OS * *ITT Population:* niraparib: 84% placebo: 77% HR, 0.70; 95% CI, 0.44 to 1.11 *HRd Population:* niraparib: 91% placebo: 85% HR, 0.61; 95% CI, 0.27 to 1.39 *HRp Population:* niraparib: 81% placebo: 59% HR, 0.51; 95% CI, 0.27 to 0.97
	Rucaparib	ATHENA-MONO/ GOG-3020/ ENGOT-ov45 [62]	High-grade epithelial histology Stage III or IV Primary CRS or Interval CRS Complete or partial response after platinum–taxane chemotherapy (bevacizumab permitted during the chemotherapy phase)	_g_*BRCA*_m_ _g_*BRCA*_wt_/HRd _g_*BRCA*_wt_/HRp	Phase 3 4:1 ratio to receive rucaparib 600 mg or placebo twice a day for up to 24 months.	*Median PFS:* *ITT Population:* rucaparib: 20.2 months placebo 9.2 months HR, 0.52; 95% CI, 0.40 to 0.68 *HRd Population:* rucaparib: 28.7 months placebo: 11.3 months HR, 0.47; 95% CI, 0.31 to 0.72 *HRp Population:* rucaparib: 12.1 months placebo: 9.1 months HR, 0.65; 95% CI, 0.45 to 0.95	OS results have not yet been reported.
	Olaparib	SOLO-1 [26,63]	High-grade epithelial histology Stage III or IV Primary CRS or Interval CRS Complete or partial response after platinum–taxane chemotherapy (bevacizumab not permitted)	_g_ *BRCA*_m_ _s_ *BRCA*_m_	Phase 3 2:1 ratio to receive olaparib 300 mg or placebo twice daily for up to 24 months.	*Median PFS:* *ITT Population:* olaparib: 56.0 months placebo: 13.8 months HR 0.33; 95% CI 0.25–0.43	*Median OS: * *ITT Population:* olaparib: Not reached placebo: 75.2 months HR, 0.55; 95% CI, 0.40 to 0.76
	Olaparib + bevacizumab	PAOLA-1 [28,64]	High-grade epithelial histology Stage III or IV Primary CRS or Interval CRS Complete or partial response after platinum–taxane chemotherapy plus bevacizumab	_g_*BRCA*_m_ _g_*BRCA*_wt_/HRd _g_*BRCA*_wt_/HRp	Phase 3 2:1 ratio to receive olaparib 300 mg or placebo twice daily for up to 24 months. Bevacizumab 15 mg/kg every three weeks was initiated with chemotherapy and continued as maintenance therapy for up to 15 months.	*Median PFS:* *ITT Population:* olaparib: 22.1 months placebo: 13.8 months HR, 0.59; 95% CI 0.49–0.72 *HRd Population:* olaparib: 37.2 months placebo: 17.7 months HR, 0.33; 95% CI 0.25–0.45 *HRp Population:* olaparib: 16.6 months placebo: 16.2 months HR, 1.00; 95% CI 0.75–1.35	*Median OS:* *ITT Population:* olaparib: 56.5 months placebo: 51.6 months HR, 0.92; 95% CI 0.76–1.12 *HRd Population:* olaparib 75.2 months placebo: 57.3 months HR, 0.62; 95% CI 0.45–0.85 *HRp Population:* olaparib: 36.8 months placebo: 40.4 months HR,1.19; 95% CI 0.88–1.63
***Recurrent Maintenance***	Niraparib	ENGOT-OV16/NOVA [23,73,74]	PSROC High-grade epithelial histology A total of ≥ 2 prior lines of platinum-based chemotherapy +/− bevacizumab	_g_*BRCA*_m_ _g_*BRCA*_wt_/HRd _g_*BRCA*_wt_/HRp	Phase 3 2:1 ratio to receive niraparib 300 mg or placebo daily until disease progression.	*Median PFS:* *_g_BRCA_m_ Population:* niraparib: 21 months placebo: 5.5 months HR, 0.27; 95% CI 0.17–0.41 *_g_BRCA_wt_*/*HRd Population:* niraparib: 12.9 months placebo: 3.8 months HR, 0.38; 95% CI 0.24–0.59 *_g_BRCA_wt_*/*HRp Population:* niraparib: 7.4 months placebo: 4.2 months HR, 0.45; 95% CI 0.34–0.61	*Median OS:* *_g_BRCA_m_ Population:* niraparib: 40.9 months placebo: 38.1 months HR, 0.85; 95% CI 0.61–1.20 *_g_BRCA_w_*_t_/*HRd Population:* niraparib: 35.6 months placebo: 41.4 months HR, 1.29; 95% CI 0.85–1.95 *OS HRp Population:* niraparib: 27.9 months placebo: 27.9 months HR, 0.93; 95% CI 0.61–1.41
	Niraparib	NORA [75]	PSROC High-grade epithelial histology A total of 2 prior lines of platinum-based chemotherapy +/− bevacizumab	_g_ *BRCA*_m_ _g_ *BRCA*_wt_	Phase 3 2:1 to receive niraparib 300 mg or placebo daily until disease progression.	*Median PFS: * *ITT Population:* niraparib: 18.3 months placebo: 5.4 months HR, 0.32; 95% CI 0.23–0.45 *_g_BRCA_m_ Population:* niraparib: Not reached placebo: 5.5 months HR, 0.22; 95% CI 0.23–0.39 *_g_BRCA_wt_ Population:* niraparib: 11.1 months placebo: 3.9 months HR, 0.40; 95% CI 0.26–0.61	*Median OS: * *ITT Population:* niraparib: 46.3 months placebo: 43.4 months HR, 0.83; 95% CI 0.56–1.21 *_g_BRCA_m_ Population:* niraparib: Not reached placebo: 47.6 months HR, 0. 77; 95% CI 0. 40–1.47 *_g_BRCA_wt_ Population:* niraparib: 43.1 months placebo: 38.4 months HR, 0.86; 95% CI 0.53–1.39
	Rucaparib	ARIEL-3 [76,77]	PSROC High-grade epithelial histology A total of ≥ 2 prior lines of platinum-based chemotherapy +/− bevacizumab	_g_*BRCA*_m_ _g_*BRCA*_wt_/HRd _g_*BRCA*_wt_/HRp	Phase 3 2:1 ratio to receive rucaparib 600 mg or placebo twice daily until disease progression.	*Median PFS: * *ITT Population:* rucaparib: 10.8 months placebo: 5.4 months HR, 0.36; 95% CI 0.30–0.45 *HRd Population:* rucaparib: 9.7 months placebo: 5.4 months HR, 0.44; 95% CI 0.29–0.66 *HRp Population:* rucaparib: 6.7 months placebo: 5.4 months HR, 0.58; 95% CI 0.40–0.85	*Median OS: * *ITT Population:* rucaparib: 36 months placebo: 43.2 months HR, 0.995; 95% CI 0.81–1.22 *HRd Population:* rucaparib: 40.5 months placebo: 47.8 months HR, 1.01; 95% CI 0.77–1.32 *HRp Population:* rucaparib: 28.6 months placebo 32.6 months HR, 1.15; 95% CI 0.78–1.70
	Olaparib	Study-19 [80,81]	PSROC High-grade serous histology A total of ≥ 2 prior lines of platinum-based chemotherapy	_g_ *BRCA*_m_ _g_ *BRCA*_wt_	Phase 2 1:1 ratio to receive olaparib 400 mg or placebo twice daily until disease progression.	*Median PFS:* *ITT Population:* olaparib: 8.4 months placebo: 4.8 months HR, 0.35; 95% CI 0.25–0.49 *gBRCAm Population:* olaparib: 11.2 months placebo: 4.3 months HR, 0.18; 95% CI 0.10–0.31 *gBRCAwt population:* olaparib: 7.4 months placebo: 5.5 months HR, 0.54; 95% CI 0.34–0.85	*Median OS:* *ITT Population:* olaparib: 29.8 months placebo: 27.8 months HR, 0.73; 95% CI 0.55–0.96 *gBRCAm Population:* olaparib: 34.9 months placebo: 30.2 months HR, 0.62; 95% CI 0.41–0.94 *gBRCAwt population:* olaparib: 24.5 months placebo: 26.6 months HR, 0.83; 95% CI 0.55–1.24
	Olaparib	SOLO2/ENGOT-Ov21 [78,79]	PSROC High-grade epithelial histology A total of ≥ 2 prior lines of platinum-based chemotherapy	_g_ *BRCA*_m_ _s_ *BRCA*_m_	Phase 3 2:1 ratio to receive olaparib 300 mg or placebo twice daily until disease progression.	*Median PFS:* *ITT Population:* olaparib: 19.1 months placebo: 5.5 months HR 0.30; 95% CI 0.22–0.41	*Median OS:* *ITT Population:* olaparib: 51.7 months placebo: 38.8 months HR 0.30; 95% CI 0.22–0.41
***Recurrent treatment***	Rucaparib	ARIEL-4 [68,69]	PSROC High-grade epithelial histology A total of ≥ 2 prior lines of chemotherapy (at least one line of PBC), no prior PARP exposure	_g_ *BRCA*_m_ _s_ *BRCA*_m_	Phase 3 2:1 ratio to receive rucaparib 600 mg twice daily or chemotherapy until disease progression.	*Median PFS:* *ITT Population:* rucaparib: 7.4 months placebo: 5.7 months HR 0.67; 95% CI 0.52–0.86	*Median OS:* *ITT Population:* rucaparib: 19.4 months placebo: 25.4 months HR 1.31; 95% CI 1.00–1.73
	Olaparib	SOLO-3 [70,71]	PSROC High-grade epithelial histology A total of ≥ 2 prior lines of chemotherapy (at least one line of PBC), no prior PARP exposure	_g_ *BRCA*_m_	Phase 3 2:1 ratio to receive rucaparib 600 mg twice daily or chemotherapy until disease progression.	*Median PFS: * *ITT Population:* 13.2 months in the olaparib group vs. 8.5 months in the placebo group (HR 0.62; 95% CI 0.43–0.91).	*Median OS:* *ITT Population:* 34.9 months in the olaparib group vs. 32.9 months in the placebo group (HR 1.07; 95% CI 0.76–1.49).

Bolded text indicates the different settings in which PARPis were used in the trials; underlined italic text highlights the trial endpoints being assessed; italicized text without underlining denotes the patient populations evaluated in the trials.

## 4. Does PARP Inhibitor Maintenance Therapy Influence Outcomes of Platinum-Based Chemotherapy Retreatment in Recurrent Disease?

As survival data from PARPi maintenance trials continue to mature, conflicting results have emerged regarding the potential benefits on OS. Some studies indicate no OS benefit following PARPi maintenance therapy, suggesting that the long-term effectiveness of these drugs may be limited. The optimal approach to managing patients who experience disease progression while undergoing PARPi treatment remains uncertain, particularly for those who progress while actively receiving a PARPi treatment. Historically, many patients with recurrent ovarian cancer have benefitted from retreatment with agents used in the first-line setting, such as carboplatin, paclitaxel, and bevacizumab [82,83]. However, one of the main concerns after the introduction of PARPis into the management of ovarian cancer is the potential for cross-resistance between PARPis and PBC, which may reduce the efficacy of subsequent treatment options in these patients. Emerging clinical data from several studies have started to explore this issue, providing evidence that progression on PARPi treatment may be associated with a poor prognosis upon retreatment with platinum-based therapies.

A multicenter study on patients with *BRCA*-mutated ovarian cancer was among the first to highlight the reduced efficacy of PBC following frontline PARPi use [84]. While the study demonstrated that olaparib could be both effective and safe as a maintenance therapy for this patient group, it also revealed a low response rate to subsequent treatments in those who progressed after maintenance. The objective response rate (ORR) was only 22.2% in those with platinum-free intervals of more than 12 months, which is approximately one-third to one-half of the rates reported in previous studies on patients who had not received PARPi treatment [84,85,86]. This finding was also noted in a secondary analysis of the SOLO2/ENGOT Ov-21 trial [87]. In this study, olaparib was administered to patients with platinum-sensitive, relapsed *BRCA*-mutated ovarian cancer who had undergone at least two prior lines of chemotherapy. As previously mentioned, the results demonstrated a significant improvement in PFS and OS with an acceptable safety profile. This secondary analysis included only patients who received chemotherapy—either PBC or non-PBC—after disease progression following treatment with olaparib or placebo. The findings revealed that the time to second progression was significantly longer in patients who had received a placebo in the SOLO2/ENGOT Ov-21 trial compared to those who had received olaparib, specifically among those who underwent post-progression PBC (HR = 2.89, 95% CI of 1.73–4.82). However, this difference was not observed in patients who received non-PBC (HR = 1.58, 95% CI of 0.86–2.90), raising questions about the role of prior PARPi exposure in the effectiveness of PBC.

Further evidence from retrospective studies has provided additional insight into this issue. In a retrospective chart review study, the outcomes of patients who received PBC after progressing on PARPi treatment were evaluated [88]. This study demonstrated that the median PFS after initiation of PBC was 219 days (interquartile range of 125–307), with the median being significantly longer in patients who had been on PARPis for more than 18 months compared to those who had been on them for 12 to 18 months and those who had received them for less than 12 months. Additionally, a retrospective international study conducted on 291 patients with ovarian cancer who were treated between 2003 and 2021 assessed the response to chemotherapy after progression on PARPi [89]. This study found that among patients with a platinum-free interval of more than six months, those who received PBC did not have worse outcomes compared to those who received non-PBC. The PBC group showed numerically better PFS, although the adjusted HR (0.68, 95% CI of 0.46–1.01) was not statistically significant (*p* = 0.0547). Another retrospective study observed an ORR of 70% in response to PBC among 10 patients who had progressed on PARPi [88].

A recently published post hoc analysis of the PAOLA-1/ENGOT-ov25 trial provided additional insights into the effectiveness of subsequent treatments in patients with recurrent ovarian cancer following PARPi therapy. This analysis specifically examined the efficacy of chemotherapy after olaparib maintenance, stratifying patients based on whether disease progression occurred during or after olaparib maintenance. Multivariate analysis revealed that the timing of progression was significantly associated with the duration between the first and second subsequent treatments, both in the overall cohort and in the subgroup of patients who received PBC and PARPi retreatment after progression. A detailed summary of studies evaluating the impact of prior PARPi treatment on the efficacy of PBC retreatment after progression is provided in Table 2.

These findings collectively do not support the hypothesis that prior exposure to PARPis may negatively influence the response to subsequent PBC. However, the variability in outcomes across different studies underscores the complexity of this relationship and the need for further investigation. Different studies have assessed this issue using varied methodologies and reported different aspects of this issue. Some studies focused on the impact of prior PARPi exposure by evaluating objective response rates (ORR) to PBC, while others examined PFS or OS as key endpoints. Additionally, the studies differed in their inclusion criteria, with some exclusively analyzing *BRCA*-mutated populations, while others included patients with both *BRCA*-mutated and wild-type tumors. Differences in the duration of PARPi exposure, timing of disease progression, number of prior lines of retreatment, and platinum-free intervals further complicate direct comparisons across studies. Additionally, the type of platinum regimen used post-PARPi progression varies between studies, which may influence the observed outcomes. Some analyses suggest a detrimental effect of prior PARPi treatment on PBC efficacy. In contrast, others indicate that specific subgroups, particularly those with longer platinum-free intervals, may still benefit from PBC retreatment. Given these discrepancies, more prospective, well-controlled studies are necessary to clarify the impact of prior PARPi exposure on PBC retreatment outcomes.

## 5. Is Retreatment with PARP Inhibitors a Viable Strategy After Prior Maintenance Failure?

Similar to the challenges and uncertainties surrounding the effectiveness of PBC retreatment following progression on PARPi, the potential clinical benefit of PARPi retreatment after prior progression on PARPi maintenance remains an open question.

Early investigations into PARPi retreatment have yielded mixed results. A secondary analysis of the QUADRA trial, conducted by Rimel et al., specifically focused on patients with prior PARPi exposure [96]. The QUADRA trial was a multicenter, single-arm study conducted across Canada and the United States, evaluating the efficacy and safety of niraparib monotherapy in patients with ovarian cancer who had undergone three or more prior chemotherapy regimens [72]. Among the more than 450 patients enrolled, 37 had previously received a PARPi, with 33 discontinuing it due to disease progression. Within this subgroup, 35 patients had evaluable outcomes, revealing that only 3% achieved a confirmed partial response, 34% had stable disease for at least seven weeks, and a clinical benefit rate of 20% at 16 weeks. Similarly, a post hoc analysis of the Rucaparib Access Programme examined a subset of ovarian cancer patients with prior PARPi exposure, with a median of five prior treatment lines before receiving rucaparib [97]. The study found a median PFS of only 2.5 months (95% CI: 1.0–4.4). Among seven patients with accessible response data, only one achieved stable disease, while six experienced disease progression, reinforcing concerns about limited efficacy in heavily pretreated patients. Essel et al. also conducted a multicenter retrospective analysis of 22 patients with recurrent ovarian cancer who underwent PARPi retreatment [98]. No complete responses were observed in this cohort, but three patients had partial responses, and 13 achieved stable disease. Notably, all three patients with partial responses harbored *BRCA* mutations. These findings suggest that while a small subset of patients may still derive some benefit from PARPi retreatment, overall response rates remain low.

The first and only prospective study specifically designed to address the concept of PARPi retreatment was the OReO/ENGOT Ov-38 trial [99]. This trial was a randomized, placebo-controlled, double-blinded study to assess the effectiveness and safety of maintenance olaparib on both *BRCA*-mutated and non-*BRCA*-mutated patient cohorts with recurrent ovarian cancer who have previously received PARPi treatment and remained platinum-sensitive to their most recent PBC. The study demonstrated that olaparib retreatment was associated with a 43% lower hazard of disease progression or death among patients with *BRCA* mutations (HR = 0.57, 95% CI of 0.37–0.87) and a 57% lower hazard among those without *BRCA* mutations (HR = 0.57, 95% CI of 0.37–0.87) with no additional safety concerns reported. An unexpected observation was the comparable or slightly greater benefit seen in the non-*BRCA*-mutated cohort. This contrasts with previous studies in the PARPi-naïve setting, where *BRCA*-mutated tumors have consistently demonstrated greater sensitivity to PARPi therapy [23,24,80].

A retrospective study involving 201 patients with recurrent ovarian cancer was conducted to evaluate the efficacy and safety of PARPi retreatment following prior failure and to compare its outcomes with chemotherapy alone [100]. The study demonstrated that patients who underwent PARPi retreatment had a significantly lower progression rate (66.7% vs. 84.0%) and a significantly longer median PFS (10.8 vs. 5.0 months) than those who received chemotherapy alone. Furthermore, the efficacy of PARPi retreatment was notably higher in patients who had achieved a complete response to their most recent therapy compared to those with a partial response. Additionally, the researchers conducted molecular analyses, which revealed that specific mutations were associated with significantly longer PFS following PARPi re-treatment, suggesting molecular profiling may enhance patient selection and improve the clinical utility of PARPi rechallenge in recurrent ovarian cancer.

## 6. Challenges and Future Directions

Resistance to PARPis remains a significant hurdle in the treatment of ovarian cancer, prompting extensive research into novel therapeutic targets and combination strategies. Several molecular pathways have been identified as contributors to PARPi resistance, and clinical trials are actively evaluating targeted treatments to counteract these mechanisms [101]. Among the promising agents being tested in combination with PARPi are phosphatidylinositol-3-kinase (PI3K) inhibitors, including alpelisib (EPIK-O/ENGOT-ov61 trial, NCT04729387) and copanlisib (NCT03586661); MEK1/2 inhibitors such as selumetinib (NCT03162627); and ATR inhibitors like ceralasertib. Tyrosine kinase inhibitors (TKIs) under investigation include bevacizumab (KGOG 3056/NIRVANA-R trial, NCT04734665; MITO25 trial, NCT03462212) and anlotinib (NCT04566952). Immune checkpoint inhibitors are also being explored in combination with PARPi, including pembrolizumab (MK-7339-001/KEYLYNK-001/ENGOT-ov43/GOG-3036 trial, NCT03740165), dostarlimab (NItCHE/MITO trial, NCT04679064), durvalumab (NCT02953457), tremelimumab (NCT04034927), and TSR-042 (anti-PD-1, NCT04673448). Other investigational therapies combined with PARPi include the histone deacetylase (HDAC) inhibitor belinostat (NCT04703920) and the nanoparticle-drug conjugate (NDC) EP0057, a cyclodextrin-based polymer backbone linked to camptothecin (EP0057-201 trial, NCT04669002). Additionally, various combination regimens are being tested, such as cediranib with durvalumab (NCT02484404), pembrolizumab with bevacizumab (SaINT-ov02 trial, NCT05158062), and dostarlimab with bevacizumab (OPAL trial, NCT03574779). These trials aim to enhance the efficacy of PARPi by targeting key resistance mechanisms. An overview of some of these important ongoing trials with their primary endpoint is provided in Table 3.

Encouraging data from some of these trials suggest that certain combination strategies may provide significant benefits. The DUO-O trial, which investigated the addition of bevacizumab, durvalumab, and olaparib to PBC in frontline maintenance therapy, has shown a notable improvement in PFS compared to bevacizumab and PBC in the interim analysis [102]. However, a critical limitation of this study is the absence of a control arm including a PARP inhibitor, reflecting the rapid evolution of treatment paradigms and the challenges in designing trials that remain relevant amid changing standards of care. Other recently published studies have also reported promising outcomes. The NCT04517357 trial demonstrated the superiority of apatinib combined with fuzuloparib over fuzuloparib alone, with a higher ORR [103]. Similarly, pembrolizumab in combination with olaparib (NCT04417192) exhibited an ORR of 70%, while the OPEB-01 trial (NCT04361370), evaluating the triplet regimen of olaparib, pembrolizumab, and bevacizumab, reported an impressive PFS rate of 88.6% at six months [104]. Additionally, findings from the PETRA trial assessed the first-in-class PARP1-selective inhibitor saruparib (AZD5305) in patients with advanced solid tumors harboring BRCA1/2, PALB2, or RAD51C/D mutations [105]. This phase I study demonstrated promising anticancer activity with a favorable safety profile. Several other combination strategies have also shown potential. The CAPRI trial (NCT03462342), a phase 2 study evaluating the ATR inhibitor ceralasertib with olaparib, found the combination to be both well-tolerated and effective [106]. Meanwhile, the EFFORT trial (NCT03579316) investigated the Wee1 inhibitor adavosertib alone and in combination with olaparib in patients with PARPi-resistant ovarian cancer, demonstrating notable efficacy in this challenging patient population [107].

Conversely, not all trials have produced favorable results. The ICON-9 trial (NCT03278717) found that cediranib combined with olaparib did not confer superior efficacy over olaparib alone in terms of PFS and OS in patients with relapsed ovarian cancer following response to PBC [108]. Similarly, the NRG-GY005 trial (NCT02502266), which compared cediranib and olaparib combinations with olaparib or cediranib alone or with standard-of-care chemotherapy, failed to demonstrate a PFS benefit over each of these arms [109]. Furthermore, the ENGOT-OV41/GEICO 69-O/ANITA trial (NCT03598270), which explored the addition of atezolizumab to chemotherapy and maintenance niraparib in recurrent ovarian cancer, did not yield significant improvements in PFS or ORR [110]. Additionally, a trial investigating durvalumab combined with olaparib and/or cediranib found that none of the experimental arms outperformed standard-of-care treatment in terms of PFS [111]. These results underscore the complexity of overcoming drug resistance and highlight the necessity of more refined therapeutic strategies.

A critical challenge in the current adoption of these agents in the management of ovarian cancer is the lack of optimal patient selection criteria. While *BRCA* mutations and HRD status are key biomarkers for predicting PARPi response, they are not always reliable indicators of long-term efficacy. Many patients without *BRCA* mutations or HRD positivity still exhibit responses to PARPi, whereas some *BRCA*-mutated or HRD-positive cases fail to derive sustained benefit. This highlights the need for more precise genomic and molecular signatures to refine patient stratification and guide treatment decisions. Ongoing research explores alternative predictive biomarkers, such as alterations in RAD51 foci formation, replication stress markers, and transcriptomic profiles, to enhance the ability to tailor PARPi-based therapies to individual patients. The toxicity and tolerability of PARPi-based combination regimens present another major hurdle. While these combinations have shown promise in improving efficacy, they often exacerbate treatment-related adverse effects, including myelosuppression (anemia, neutropenia, thrombocytopenia), gastrointestinal toxicity (nausea, vomiting, diarrhea), and fatigue. These toxicities can limit the duration of therapy and reduce patients’ quality of life, necessitating careful dose optimization, supportive care strategies, and proactive management of side effects to maximize therapeutic benefits without compromising tolerability.

Furthermore, the optimal sequencing and integration of PARPi with other treatment modalities remain unresolved. The interplay between PARPi, chemotherapy, immunotherapy, and other targeted agents is complex, and their sequential or concurrent administration can influence both efficacy and resistance mechanisms. For instance, while combining PARPi with immune checkpoint inhibitors holds potential due to PARPi-induced genomic instability and enhanced neoantigen exposure, the timing and dosing of this combination remain areas of active investigation. Similarly, integrating anti-angiogenic agents, ATR inhibitors, and MEK inhibitors with PARPi requires careful consideration of pharmacokinetics, toxicity profiles, and potential antagonistic interactions. Compounding these challenges is that current clinical trial findings on these combination strategies remain conflicting, as mentioned above.

A major barrier to defining optimal treatment strategies is the significant heterogeneity in studies evaluating PARPi-based management. Differences in study designs, treatment arms, primary and secondary endpoints, and patient population characteristics create challenges in drawing consistent conclusions across trials. Additionally, the retrospective nature of several studies, small sample sizes, and variations in prior treatment exposures further complicate the interpretation of results and the establishment of standardized treatment guidelines. To address these challenges, future research should focus on prospective, randomized trials with standardized inclusion criteria, comprehensive biomarker analyses, and long-term follow-up data. Additionally, real-world evidence and large-scale registries may provide valuable insights into the effectiveness and safety of PARPi-based combinations outside controlled clinical settings. As our understanding of resistance mechanisms, patient selection strategies, and treatment sequencing evolves, these efforts will be crucial in optimizing the use of PARPi in ovarian cancer management.

## 7. Conclusions

In conclusion, while PARPi therapy has revolutionized the treatment of ovarian cancer, significant challenges remain. Drug resistance, patient selection, toxicity management, therapeutic sequencing, and heterogeneity in the current body of evidence all contribute to the complexity of optimizing PARPi use. Continued research and innovative clinical trial designs are essential to address these limitations and develop more effective, personalized strategies for patients. The future of ovarian cancer treatment will likely depend on a multifaceted approach that integrates genomic profiling, novel drug combinations, and adaptive treatment strategies to enhance long-term outcomes.

## Figures and Tables

**Table 2:** Studies evaluating the effects of PARP inhibitor maintenance therapy on sensitivity to subsequent lines of platinum-based chemotherapy.

First Author	Country	Study Design	Number of Participants	Types of Ovarian Cancer	PARPi Administered	Findings
Cecere et al. [84]	Italy	Retrospective study	234	*BRCA*-mutated recurrent platinum-sensitive	Olaparib	Post-progression response to PBC in terms of ORR of 22.2% in those who had a platinum-free intervals of more than 12 months.
Frenel et al. [87]	International	Posthoc analysis on an RCT	96	*BRCA*-mutated recurrent platinum-sensitive	Olaparib	Significantly longer time to a second progression after post-progression PBC initiation in those who have received a placebo compared to those who received olaparib.
Rose et al. [90]	USA	Retrospective study	115	*BRCA*-mutated recurrent platinum-sensitive	Various PARPis (niraparib, olaparib, rucaparib, and veliparib)	Following each of the second and third courses of PBC, patients who had not been exposed to PARPi had significantly longer PFS than those with no prior exposure.
Park et al. [91]	Republic of Korea	Retrospective study	197	*BRCA*-mutated recurrent platinum-sensitive	Olaparib	Post-progression chemotherapy, either PBC or non-PBC, was associated with a shorter PFS in those who received prior olaparib maintenance therapy.
Baert et al. [92,93]	Germany	Retrospective study	92	Recurrent	Olaparib and niraparib	Prior PARPi treatment negatively reduced the effectiveness of later PBC, evidenced by a higher progression rate in the PARPi group (40% vs. 9% in the control group, *p* = 0.003).
Plaja Salarich et al. [93]	Spain	Retrospective study	54	Recurrent	PARPi	An overall ORR of 33.3% was observed for subsequent PBC, increasing to 42.9% in patients with a platinum-free interval of over 12 months.
Gadducci et al. [94]	Italy	Retrospective study	103	Recurrent platinum-sensitive	Various PARPis (olaparib, niraparib, and rucaparib)	Subsequent chemotherapy, PBC and non-PBC combined, was associated with an ORR of 41.9% in those who had platinum-free interval of more than 12 months.
Romeo et al. [95]	Spain	Retrospective study	74	Platinum-sensitive recurrent	Various PARPis (olaparib, niraparib, and rucaparib)	Subsequent PBC after PARPi was associated with an ORR of 41.9% and a median PFS and OS of 6.6 and 20.6 months, respectively.
Dugan et al. [88]	USA	Retrospective study	40	Platinum-sensitive recurrent	Various PARPis (olaparib, niraparib, and rucaparib)	Median PFS after starting PBC was 219 days (IQR 125–307), significantly longer in those on PARPis for over 18 months.
Xu-Vuillard et al. [89]	International	Retrospective study	291	Recurrent	PARPis	Patients with a platinum-free interval over six months had better PFS with PBC than non-PBC, though not statistically significant (HR = 0.68, 95% CI of 0.46–1.01).
Nakao et al. [88]	Japan	Retrospective study	10	Recurrent	Olaparib and niraparib	Post-progression response to PBC showed an ORR of 70%, with all patients having a platinum-free interval of more than six months.

**Table 3:** Ongoing clinical trials evaluating combination strategies with PARP inhibitors in ovarian cancer.

NCT Number	Study Title	Phase	Location	Primary Endpoint	Targeted Additional Mechanism
NCT04729387	Alpelisib Plus Olaparib in Platinum-resistant/Refractory, High-grade Serous Ovarian Cancer, with no Germline *BRCA* Mutation Detected	3	Australia, Austria, Belgium, Brazil, Canada, China, Czechia, Denmark, Finland, France, Germany, Italy, the Republic of Korea, Malaysia, Mexico, the Netherlands, Portugal, Russia, Singapore, Slovakia, Spain, Taiwan, Turkey, the United Kingdom, and the United States of America	Progression-free survival	The phosphoinositide 3-kinase pathway inhibition
NCT04679064	Trial on Niraparib-TSR-042 (Dostarlimab) vs. Physician’s Choice Chemotherapy in Recurrent, Ovarian, Fallopian Tube, or Primary Peritoneal Cancer Patients Not Candidate for Platinum Retreatment	3	Italy	Overall survival	Programmed cell death protein 1 blockade
NCT03740165	Study of Chemotherapy with Pembrolizumab (MK-3475) Followed by Maintenance with Olaparib (MK-7339) for the First-Line Treatment of Women with BRCA Non-mutated Advanced Epithelial Ovarian Cancer (EOC) (MK-7339-001/KEYLYNK-001/ENGOT-ov43/GOG-3036)	3	Australia, Belgium, Brazil, Canada, Chile, Colombia, Czechia, France, Germany, Hungary, Israel, Italy, Japan, the Republic of Korea, Poland, Russia, South Africa, Spain, Taiwan, Turkey, Ukraine, and the United States of America	Progression-free survival	Programmed cell death protein 1 blockade
NCT04734665	Niraparib and Bevacizumab Maintenance Therapy in Platinum-sensitive Recurrent Ovarian Cancer Patients Previously Treated with a PARP Inhibitor	2	Republic of Korea	6-month progression-free survival rate	Vascular endothelial growth factor A inhibition
NCT04669002	EP0057 in Combination with Olaparib in Advanced Ovarian Cancer	2	Hungary, the United Kingdom, and the United States of America	Objective response rate	Topoisomerase I inhibition
NCT04566952	Anlotinib Combined with Dose-reduced Olaparib in Patients with Platinum-Sensitive Recurrent Ovarian Cancer	2	China	Progression-free survival, adverse events	Inhibition of vascular endothelial growth factor receptors, platelet-derived growth factor receptors, fibroblast growth factor receptors, c-Kit, and rearranged during transfection
NCT04556071	Efficacy and Safety of Niraparib Combined with Bevacizumab in Platinum Refractory/Resistant Recurrent Ovarian Cancer	2	China	Objective response rate	Vascular endothelial growth factor A inhibition
NCT05158062	Pembrolizumab and Bevacizumab with Chemotherapy Followed by Pembrolizumab, Bevacizumab and Olaparib in Recurrent Ovarian Cancer	2	Japan	Two-year progression-free survival rate	Vascular endothelial growth factor A inhibition and programmed cell death protein 1
NCT03574779	A Study to Evaluate the Efficacy and Safety of Novel Treatment Combinations in Participants with Ovarian Cancer (OPAL)	2	Canada, Spain, Turkey, and the United States of America	Objective response rate	Vascular endothelial growth factor A inhibition
NCT02953457	Olaparib, Durvalumab, and Tremelimumab in Treating Patients with Recurrent or Refractory Ovarian, Fallopian Tube or Primary Peritoneal Cancer with BRCA1 or BRCA2 Mutation	2	United States of America	Dose-limiting toxicities, 3- and 6-month progression-free survival	Cytotoxic T-lymphocyte–associated protein 4 blockade and programmed death ligand 1 blockade
NCT04034927	Testing the Addition of an Immunotherapy Drug, Tremelimumab, to the PARP Inhibition Drug, Olaparib, for Recurrent Ovarian, Fallopian Tube or Peritoneal Cancer	2	United States of America	Progression-free survival, dose-limiting toxicities	Cytotoxic T-lymphocyte–associated Protein 4 blockade
NCT02484404	Phase I/II Study of the Anti-Programmed Death Ligand-1 Durvalumab Antibody (MEDI4736) in Combination with Olaparib and/or Cediranib for Advanced Solid Tumors and Advanced or Recurrent Ovarian, Triple Negative Breast, Lung, Prostate and Colorectal Cancers	1–2	United States of America	Maximum tolerated dose, objective response rate	Programmed death ligand 1 and vascular endothelial growth factor receptor 1, receptor 2, and receptor 3 inhibition
NCT02571725	PARP-inhibition and CTLA-4 Blockade in BRCA-deficient Ovarian Cancer	1–2	United States of America	Maximum tolerated dose, objective response rate	Cytotoxic T-lymphocyte–associated protein 4 blockade
NCT03462212	Carboplatin-Paclitaxel-Bevacizumab vs. Carbo-Pacli-Beva-Rucaparib vs. Carbo-Pacli-Ruca, Selected According to HRD Status, in Patients with Advanced Ovarian, Primary Peritoneal and Fallopian Tube Cancer, Preceded by a Phase I Dose Escalation Study on Ruca-Beva Combination	1–2	Italy	Maximum tolerated dose, progression-free survival	Vascular endothelial growth factor A inhibition
NCT04703920	Talazoparib in Combination with Belinostat for Metastatic Breast Cancer, Metastatic Castration-Resistant Prostate Cancer, and Metastatic Ovarian Cancer	1	United States of America	Dose-limiting toxicities	Histone deacetylase inhibition
NCT03162627	Selumetinib and Olaparib in Solid Tumors	1	United States of America	Maximum tolerated dose	The mitogen-activated protein kinase kinase 1 and 2 pathway inhibition
NCT04673448	Niraparib and TSR-042 for the Treatment of *BRCA*-Mutated Unresectable or Metastatic Breast, Pancreas, Ovary, Fallopian Tube, or Primary Peritoneal Cancer	1	United States of America	Best objective response	Programmed cell death protein 1 blockade
NCT03586661	Niraparib and Copanlisib in Treating Patients with Recurrent Endometrial, Ovarian, Primary Peritoneal, or Fallopian Tube Cancer	1	United States of America	Maximum tolerated dose	The phosphoinositide 3-kinase pathway inhibition

## Data Availability

No new data were created or analyzed in this study. Data sharing is not applicable to this article.
